# Through the Prism: Shining Light on LGBTQIA+ Applicant Identities and Influences

**DOI:** 10.5811/westjem.50598

**Published:** 2026-05-18

**Authors:** Kayla Iuliucci, Lea Moujaes, David Rudolph, Peter Fredericks, Blake Denley, Samuel Paskin, Arlene S. Chung, Jaime Jordan, Edgardo Ordonez, Laura Smylie, Simiao Li-Sauerwine, P. Logan Weygandt

**Affiliations:** * Johns Hopkins University School of Medicine, Department of Emergency Medicine, Baltimore, Maryland; † Ochsner Health, Department of Emergency Medicine, New Orleans, Louisiana; ‡ Larner College of Medicine at the University of Vermont, Department of Emergency Medicine, Burlington, Vermont; § Oregon Health & Science University, Department of Emergency Medicine, Portland, Oregon; ¶ The Ohio State University College of Medicine, Department of Emergency Medicine, Columbus, Ohio; || Baylor College of Medicine, Department of Emergency Medicine, Houston, Texas; # Wayne State University School of Medicine, Department of Emergency Medicine, Detroit, Michigan

## Abstract

**Introduction:**

Program diversity impacts rank-list creation for emergency medicine (EM)-bound applicants, but how lesbian, gay, bisexual, transgender, queer or questioning, intersex, asexual, and other sexual and gender minorities (LGBTQIA+) identities influence residency selection is unknown. This study investigates general patterns in EM applicant LGBTQIA+ identities, disclosure of those identities, and how LGBTQIA+ factors impact residency selection. Additionally, we present data exploring the relationship between medical school location and the location of top-ranked programs for LGBTQIA+ and non-LGBTQIA+ identifying applicants.

**Methods:**

We surveyed 2,287 EM-bound United States MD/DO applicants who applied to one of five author EM programs and programs affiliated with the Emergency Medicine Education Research Alliance from May 16–June 30, 2024. The survey included multiple-choice, free-text, and Likert scale questions. Data were explored with descriptive statistics, and we used chi-square and Fisher exact tests to compare differences in proportions. We also analyzed applicants’ medical school state and a graphical representation of the top three positions on their residency rank lists, using inverse-proportional weighting.

**Results:**

Of 445 respondents (19.4%), 59 (13.3%) identified as LGBTQIA+. Gender identities included 173 cisgender men (38.9%), 254 cisgender women (57.1%), one transgender man (0.2%), one transgender woman (0.2%), four non-binary (0.9%), one genderqueer (0.2%), and seven “preferred not to answer” (1.6%). Among LGBTQIA+ respondents, seven (11.9%) disclosed their status within the application, nine (15.3%) during the interview, 18 (30.5%) in both, and 25 (42.4%) did not disclose. Among 56 respondents, 36 (64.3%) supported adding LGBTQIA+ status to the residency application; 20 (35.7%) did not. Of the program factors considered, program diversity (91.1%) and commitment to underserved communities (96.4%) were significantly more important for LGBTQIA+ respondents (*P* < .01), while proximity to partner(s) (64.3%; *P* < .01) and program length (66.1%; *P* = .02) were significantly less important compared with non-LGBTQIA+ respondents. Additional factors that influenced LGBTQIA+ applicants’ rank list included political environment, friendliness of the learning environment, and presence/absence of anti-LGBTQIA+ laws.

**Conclusion:**

Many LGBTQIA+ applicants do not disclose their identities when applying for residency. LGBTQIA+ respondents value program diversity and commitment to underserved communities, and they consider LGBTQIA+-specific factors such as the presence of anti-LGBTQIA+ legislation. These insights can inform residency programs and recruitment practices.

## INTRODUCTION

Diverse physicians enhance team dynamics, patient outcomes, and access to care for underserved communities.^1^ Although there has been increasing focus on diversity of trainees in medical education, sexual and gender diversity has not received the same emphasis.^2^ Within emergency medicine (EM), increasing lesbian, gay, bisexual, transgender, queer or questioning, intersex, asexual, and other sexual and gender minorities (LGBTQIA+) diversity is critical, as LGBTQIA+ patients frequently rely on emergency departments (ED) due to barriers to primary care and face significant health disparities. Patient concerns about discrimination and clinician bias can lead to ED avoidance and delays in seeking care for urgent or emergent medical needs.^3^ This necessitates understanding LGBTQIA+ applicant decision-making and reflecting on residency recruitment practices.

Unlike other minority populations, LGBTQIA+ applicants face a unique challenge: Their identity is not necessarily visible or apparent on applications or during interviews. This invisibility creates a deliberate decision about whether, when, and how to “come out” during the application process while weighing competing priorities such as successfully matching, identifying optimal training, considering geographic preferences, and assessing LGBTQIA+ friendliness.

Previous research found that both program factors and geographic location were important influences on medical students’ selection of EM residencies.^4^ A more recent study evaluating influences on under-represented in medicine (URiM) applicants to EM highlighted the importance of program diversity in residency selection.^5^ However, it remains unclear whether these findings would apply to LGBTQIA+ applicants given the challenges of identity invisibility and choice to disclose. Progress has been made in LGBTQIA+ education in EM programs, with 75% now including content on LGBTQIA+ health.^6^ However, curricular enhancements alone may not suffice in creating environments where LGBTQIA+ applicants feel supported. Implicit bias, microaggressions, and the lack of visible representation continue to create barriers, potentially leading to career dissatisfaction and higher turnover rates among LGBTQIA+ physicians.^7^

Our primary objective was to determine factors that influence medical students’ ranking of EM residency programs. The secondary objectives were to assess whether factors influencing program ranking differ between LGBTQIA+ and non-LGBTQIA+ applicants, and to explore the impact of LGBTQIA+–specific applicant choices, and how applicants disclose LGBTQIA+ status during the residency selection process, if at all.

## METHODS

### Study Design and Population

This study was reviewed by the primary author’s institutional review board and deemed exempt. We employed a cross-sectional survey to assess the experiences and perspectives of medical students applying for first-year positions in EM residency programs in the United States. The survey was distributed to a convenience sample of applicants who had applied to author programs at the following institutions: Baylor College of Medicine; Johns Hopkins School of Medicine; Ohio State University; David Geffen School of Medicine at the University of California Los Angeles; and Wayne State University/Detroit Receiving Hospital. We recruited participants in accordance with the American Association of Medical Colleges policy, which permits the use of Electronic Residency Application Service applicant data by programs for research purposes.^8^ We included medical students from U.S. medical schools who were applying for a first-year categorical position with one or more of the EM residency programs listed above. International medical graduates were excluded.

**Population Health Research Capsule**

What do we already know about this issue?

*Program diversity influences emergency medicine (EM) residency selection, yet the impact of LGBTQIA+ identity on applicant decision-making remains understudied*.

What was the research question?

*How do LGBTQIA+ identity, disclosure, and related factors influence the rank list decisions of applicants to EM residency?*

What was the major finding of the study?

*LGBTQIA+ applicants more often valued program diversity (91.1% vs 72.2%) and commitment to the underserved (96.4% vs 81.0%; P < .01)*.

How does this improve population health?

*Understanding LGBTQIA+ applicant priorities can guide inclusive recruitment practices and strengthen workforce diversity to better serve marginalized populations*.

### Setting

We distributed the survey via Qualtrics vXM 2024 (Qualtrics International, Inc, Provo, UT) from May 16–June 30, 2024. We offered potential subjects the chance to win one of eight gift cards ($50) in appreciation of their participation. We de-identified all data and stored it on a secure server. We sent three reminder emails at regular intervals to non-responders. Response rate was calculated using the American Association for Public Opinion Research’s Response Rate 2 definition.^9^

### Instrument Development

Our survey development and reporting approach was informed by guidelines for survey research methodology, and we employed Messick’s framework as our validity model.^10^,^11^ Our CROSS checklist is included in Supplement 1. A diverse group of experts developed the survey based on prior literature and the authors’ personal experiences.^4^,^5^,^12^ We used a modified Delphi approach to further enhance content validity.^13^ We piloted the survey with 13 first-year residents from the primary author’s institution to provide response process validity evidence, and we modified items for clarity based on pilot feedback. As we were targeting a similar population, general demographic questions (Q2–9) were adapted from Weygandt et al, with expansion on the gender identity question.^5^ We retained the general diversity question from prior surveys to capture the broader importance of diversity, recognizing that applicants hold intersecting identities.^4^,^5^,^12^ Question 17 examined specific LGBTQIA+-related factors influencing program selection. Questions (Q11, 18, and 19) regarding factors that influence applicants were adapted from Love et al, including geographic location, proximity to family and significant others, cost of living, interview experience, program length, personal experiences with residents and faculty, program type, program reputation, and diversity within the program.^4^ New questions were added to determine ranked program locations and patterns of LGBTQIA+ status disclosure. The final survey consisted of 21 questions, two of which were matrices assessing program and LGBTQIA+ factors (Supplement 2). It included multiple-choice, Likert scale, and free-text items.

### Measurements/Key Outcome Measures

We collected standard demographic data including age, location, and marital status, as well as specific demographic data about LGBTQIA+ identities and URiM identities. Marital status and non-traditional status of the applicant were included as an extension of prior work by Weygandt et al,^5^ determining whether there were different trends for LGBTQIA+ vs non-LGBTQIA+ applicants.

### Data Analysis

We used Stata SE v18^14^ (StataCorp, LLC, College Station, TX) and R statistical software (The R Foundation for Statistical Software, Vienna, Austria).^15^ We employed simple descriptive statistics, including proportions and measures of central tendency. After analysis of the histogram of distribution of responses, to simplify data interpretation we dichotomized the 5-point Likert scales for residency choice factors between LGBTQIA+ and non-LGBTQIA+, grouping “extremely important,” “very important,” and “moderately important” as “important” and “slightly important” and “not at all important” as “not important.” The original response distributions prior to dichotomization are available in Supplement 3: importance of LGBTQIA+-related factors for rank-list creation, and Supplement 4: comparison of residency factors between LGBTQIA+ and non-LGBTQIA+ respondents. We used the chi-square and Fisher exact tests, when appropriate, to compare differences in proportions.

We conducted a nonresponse bias analysis using wave analysis, as data for nonrespondents were unavailable; the institutional review board (IRB) only permitted analysis of respondents who consented and completed the survey. Wave analysis can be used when there is no available data for nonrespondents with the logic that late respondents can be used as a surrogate for nonrespondents given the delay in taking the survey. The initial survey was disseminated on May 16, 2024, with subsequent reminders sent on May 28, June 10, June 17, and June 24. We performed comparisons in the wave analysis between respondents who completed the survey after the first dissemination (May 16) compared to the need for a reminder (after May 28).^16^

We employed *tidyverse* and *sf*^17^ packages within R statistical software to visualize state-level geographic data. We chose state-level analysis over regional divisions due to the heterogeneous representation of LGBTQIA+ applicants across regions; state-level examination allowed greater granularity and accuracy, given that not all U.S. states were represented by LGBTQIA+ respondents. Our central hypothesis was that graduating medical students, especially those identifying as LGBTQIA+, consider state-level legislative environments around LGBTQIA+ issues when ranking residency programs to a greater extent than non-LGBTQIA+ applicants. Because relevant legislative influences often operate at the state rather than the regional level, this approach supports a more precise analysis. These data included the location of an individual’s medical school and a graphical representation of the top three positions on their residency rank lists.

We used each respondent’s rank order to calculate an inversely proportional weighted (IPW) rank. The use of IPW was motivated by the assumption that applicants place more weight on their highest ranked program, optimizing sensitivity in measuring their true preferences. The IPW enables us to reflect that first-choice programs are prioritized above second and third choices, rather than attributing equal value to all top selections as would occur with unweighted counts. Our weighted ranking schema followed the principles of IPW, giving greater weight to higher ranks while assigning less weight to lower ranks to provide a more practical and holistic view of a student’s rank choices. We assigned a weight of 0.5 to each first rank (#1 position on rank list), 0.33 to a respondent’s second rank (#2 position on rank list), and 0.16 to the third rank (#3 position on rank list).

Missing responses were assigned to the next highest ranked state. For example, if an applicant’s second-ranked residency state was missing, we used their first-ranked state in its place for analysis. Proportions were calculated for state of medical school attendance, inversely weighted rank, and the difference between the two.^17^,^18^ To determine directionality, we calculated the difference in proportions between inversely weighted ranks and state of medical school attendance to identify intended geographic movement based on rank lists. We also hypothesized that there might be differences based on LGBTQIA+ identity and stratified accordingly.

## RESULTS

Of 2,287 EM-bound applicants to our institutions, 445 (19.5%) responded to the survey. The age in years of respondents ranged from 24–49, with a mean of 28.5 (standard deviation 3.1). We report the characteristics of participants in Table 1.

### Ranking Factors for LGBTQIA+ Identifying Applicants to Emergency Medicine

The importance of program factors to ranking decisions is displayed in Table 2. We collapsed the 5-point Likert scale as follows. “extremely important,” “very important,” and “moderately important” were considered “important” while “slightly important” and “not at all important” were considered as “not important.”

**Figure 1:**
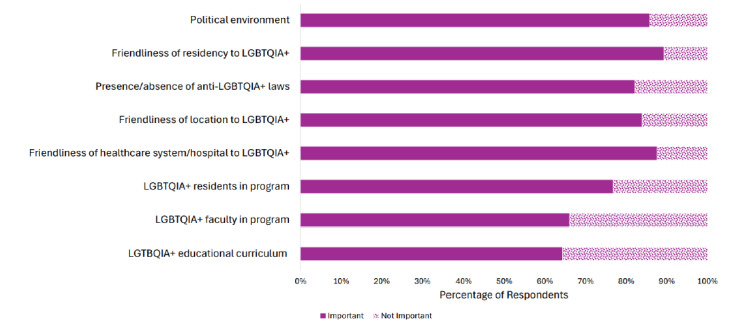
LGBTQIA+-specific factors that influence the rank lists of emergency medicine residency applicants’ (N = 56): dichotomized responses (important vs not important).

Proximity to partner (*P* < .01) and program length (*P* = .02) were significantly less important for LGBTQIA+ respondents while diversity within the program (*P* < .01) and program commitment to the underserved (*P* <.01) were significantly more important for LGBTQIA+ respondents compared to non-LGBTQIA+ respondents. There were no statistically significant differences between LGBTQIA+ and non-LGBTQIA+ applicants in the importance they placed on the other factors (*P* > .05). Factors important to LGBTQIA+ identifying applicants are displayed in Figure 1.

### Nonresponse Bias Wave Analysis

A total of 180 respondents responded to the first dissemination of the survey compared to 265 who responded after a reminder email. There were no statistically significant differences between early vs late respondents for demographic variables of gender, marital status, race, ethnicity, and sexual orientation. (See Supplement 5, Table 1.) Age distribution differed significantly between early and late respondents (Wilcoxon rank-sum test, *P* = .02), although the median age (28 years) was the same in both groups. There were no statistically significant interactions between LGBTQIA+ status and time of survey completion (early vs late respondents) for the residency selection factors in Table 2 (Supplement 5, Table 2).

### Disclosure of LGBTQIA+ Status

Of 59 respondents, 7 (11.9%) disclosed LGBTQIA+ status in their application, 9 (15.3%) in their interview, 18 (30.5%) in both, and 25 (42.4%) did not disclose. In the application, 8 respondents disclosed via their pronouns, 14 via their personal statement, 18 via experiences, 4 via hobbies, and 1 via demographics. One applicant disclosed in the context of couples matching with their partner. During the interview process, 28 (47.5%) applicants did not disclose, 20 (33.9%) did at some interviews, and 11 (18.6%) did at all interviews. When asked about adding LGBTQIA+ status to the standard residency application, 64.3% were in favor while 35.7% were not.

### Geographic Distribution of Applicants

**Figure 2:**
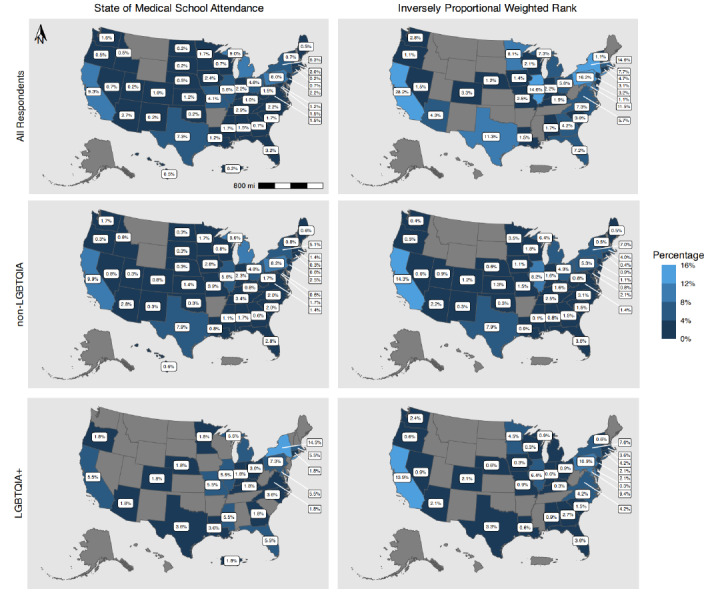
Geographic distribution of survey respondents by location of medical school and inversely proportional weighted ranks stratified by LGBTQIA+ status.

The geographic distribution of survey respondents’ medical schools is represented in Figure 2.

Of the 445 survey respondents, 22 did not provide rank list preferences and 13 did not respond to the sexual orientation and gender identity questions; therefore, both were removed from the analysis for a total of 410 respondents in the maps.

The top three states of medical school attendance for all applicants were California (9.3%), Michigan (9.0%), and Pennsylvania (8.0%). Of the LGBTQIA+ respondents, 14.5% attended medical school in New York and 7.3% in Pennsylvania. California totaled 28.2% of all IPW ranks, followed by 16.2% for residencies in Pennsylvania and 14.6% for residencies in New York and Illinois. Of LGBTQIA+ survey respondents, 13.9% of the IPW ranks were for programs located in California, 10.9% for Pennsylvania, 9.4% for Maryland, and 7.6% for New York.

**Figure 3:**
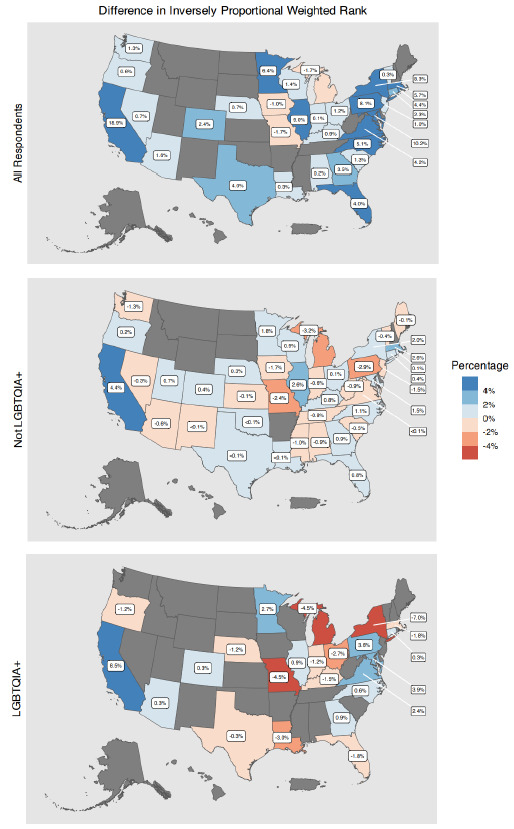
Geographic distribution of difference between inversely proportional weighted ranks and state of medical school attendance. This figure represents the difference between the first column and second column of Figure 2.

Figure 3 reflects the difference in the proportions between the IPW ranks for residency programs and the location of the survey respondents’ medical schools.

There was a positive difference of 18.9% for all respondents between the IPW ranks for residencies in California (28.2%) and the proportion of medical students attending medical school in California (9.3%). For LGBTQIA+ applicants, California had the highest positive difference of 8.5%, reflecting the difference that 5.5% of the LGBTQIA+ respondents were attending medical school in California while receiving 13.9% of all LGBTQIA+ IPW ranks. The next states with the highest positive difference were Maryland (3.9%), Pennsylvania (3.6%), Minnesota (2.7%), and Virginia (2.4%). States with the largest negative absolute differences were New York (−7.0%), Michigan (−4.5%), Missouri (−4.5%), Louisiana (−3.0%), and Ohio (−2.7%).

## DISCUSSION

Our analysis of LGBTQIA+ applicants provides insight into applicant decision-making and the influence of diversity factors, highlighting the need for residency programs to prioritize diversity in their recruitment strategies. For LGBTQIA+ applicants, a program’s commitment to underserved communities also played a key role in ranking decisions. While EM as a specialty already emphasizes care for underserved populations, residency programs can further demonstrate their commitment to these communities—a factor that significantly influences LGBTQIA+ applicants and other under-represented groups, including those marginalized by race and gender.^19^

When asked about specific LGBTQIA+ factors, applicants highlighted both external factors, such as the broader political environment, and internal program-specific considerations. Consistent with prior studies of URiM, LGBTQIA+ applicants in this study prioritized diverse and inclusive environments.^19^ Among external factors, political environment was the most influential factor for applicants, especially the presence or absence of state-level anti-LGBTQIA+ laws. Individuals may be reluctant to move to places where their rights may not be protected or where they could face legal discrimination, although further research is needed to fully explain the true decision-making process behind these preferences. The implications of the laws extend beyond legal protections and may reflect the values of the local community, patient population, hospital, and program itself. A supportive political climate, on the other hand, is essential for ensuring family rights, access to healthcare, freedom from violence/harassment, and freedom of assembly and expression—issues central to LGBTQIA+ advocacy and safety.^20^,^21^ The political climate of a program’s location can have profound implications for both personal safety and professional experiences.

The interaction of geography, program factors, and legislation is complex and not easily explained by region alone. Based on IPW differences, there appear to be some trends for LGBTQIA+ applicants to more highly rank residencies in states with more LGBTQIA+-friendly legislation (eg, California, Maryland, Virginia) and less likely to rank residencies in states with anti-LGBTQIA+ legislation (Florida, Louisiana, Missouri), according to the American Civil Liberties Union classification. California’s high desirability among LGBTQIA+ applicants may reflect its comprehensive legal protections for individuals, established communities in major metropolitan areas, and state-level policies supporting gender-affirming care and anti-discrimination protections. Interpretations regarding regions are difficult due to missing data and underpowered for statistically significant results. Interestingly, LGBTQIA+ trainees appear to be less often ranking New York, Massachusetts, and Oregon residency programs —the latter being particularly unexpected given Oregon’s strong LGBTQIA+ protections, suggesting that factors beyond state legislation alone influence applicant decisions.

While program-specific factors such as the presence of LGBTQIA+ residents and faculty, and an inclusive curriculum, were still important, they appear to be less important than the political environment, the friendliness of the location, anti-LGBTQIA+ laws, and the friendliness of the residency and healthcare system. This may suggest that LGBTQIA+ applicants find support outside the residency program, such as in their own social networks, family, and communities. Additionally, these applicants could be more familiar with LGBTQIA+ issues and inclusive education. Further studies are needed to clarify qualitatively the reasons for these trends in LGBTQIA+-specific factors.

A substantial portion of LGBTQIA+ applicants (42.4%) chose not to disclose their identity during the application or interview process. Of those who did disclose, more disclosed during interviews rather than through application materials such as pronouns or personal statements. Notably, while 42.4% chose not to disclose, 64.3% supported adding LGBTQIA+ status to the standard residency application. This is consistent with prior research that has shown that many LGBTQIA+ individuals are reluctant to disclose gender identity or sexual orientation in professional contexts due to concerns about discrimination.^22^,^23^ Nondisclosure may stem from fear of emotional, verbal, or physical abuse, social exclusion, workplace harassment, or job termination.^24^–^27^ The preference for disclosure during interviews suggests these provide a more personal context for gauging program inclusivity before revealing personal information.

The discordance between disclosure behavior and support for formal mechanisms may reflect concerns about the informal nature of current methods, where applicants lack control over how and when their identity becomes visible. A standardized, optional field could provide greater agency in disclosure decisions. This aligns with growing calls within medical education to enhance visibility and support for LGBTQIA+ trainees.^23^ Applicants in favor of this change indicate that many perceive advantages, such as better alignment with residency programs that prioritize diversity and inclusivity. However, there remains a substantial proportion who would prefer not to disclose, underscoring the need for further research to identify ways to enhance the ability to disclose while respecting an individual’s right to choose when, if, and how they do so.

Future research with expanded sample sizes and longitudinal data is needed to continue exploring these patterns to evaluate the impact of changes to the residency application process on their experiences. In the process of attracting greater numbers of LGBTQIA+ applicants, there are steps that residency programs can take to promote inclusivity for the LGBTQIA+ community. Methods include implementing initiatives such as confidential, one-on-one discussions between applicants and residents who identify as LGBTQIA+; hosting social events focused on diversity and inclusivity specifically for LGBTQIA+ applicants; establishing committees dedicated to advancing diversity and inclusion among faculty and trainees; and developing curricula that ensure inclusive and affirming care for sexual and gender minority patients.^28^–^31^

While programs with intentional efforts to enhance diversity and inclusion improve patient outcomes, foster innovation, and improve trainee satisfaction,^32^ there is limited research on how LGBTQIA+ representation in residency programs influences applicants’ decisions. Studies have demonstrated that pre-interview messaging, interview day experiences, and post-interview communication are critical touchpoints in attracting a diverse cohort. Challenges such as implicit bias, minority tax, and geographic misperceptions persist.^33^ Mentorship is another pivotal factor in supporting LGBTQIA+ trainees and a lack of visible mentors within programs often exacerbates feelings of isolation and exclusion.^34^ This study reinforces the significance of these factors and suggests that strengthening inclusive practices during recruitment may help attract applicants who bring diversity and perspective to EM.

## LIMITATIONS

The study has several limitations. First, we used a convenience sample of applicants to selected institutions, which limits the generalizability of the findings. While the study questionnaire was based on prior surveys in the literature, we also added elements based on the authors’ personal experiences as members of the queer community in EM. For example, we all were forced to choose where to disclose our LGBTQIA+ identities if we decided to disclose at all. We also experienced variable receptivity to those identities as we travelled across the country during interview season. While some of the authors identify as gay, lesbian, pan-sexual, bisexual, and queer, we do not represent the entire spectrum of LGBTQIA+ identities, and our perspective may, therefore, be somewhat limited.

The response rate of 19.5% is a primary limitation of this study, and only 13.3% of respondents identified as LGBTQIA+, limiting the generalizability of our findings. The overall proportion is similar to the proportion of LGBTQIA+ identifying people in the US, which is 9.3%,^35^ so we believe we have a relatively representative sample. The low absolute numbers of applicants identifying as LGBTQIA+ constrained further investigations and statistical power, such as subgroup analyses for female-identifying and URiM applicants. Our nonresponse bias analysis was limited by the lack of data on nonrespondents; consequently, we relied on wave analysis comparing early and late respondents, which did not show any statistically significant differences. This method may not detect all sources of bias, as late respondents may not be representative of nonrespondents, potentially limiting the generalizability of results. While we collected data on how and whether applicants disclosed their LGBTQIA+ identities, we did not assess whether they disclosed to all programs or only a subset. Additionally, the limited depth of the free-text responses led us to decide against a qualitative analysis. However, further studies could address these gaps through in-depth interviews or focus-group discussions to better explore trends from our quantitative data.

We also acknowledge that the surveyed sites are more academic-focused and urban programs. Although this may represent a large number of the total applicant pool, it still could affect the generalizability of these results. Additionally, the uneven distribution of EM residency programs across states may further influence our findings. Some states with higher program concentrations may be disproportionately represented in applicant considerations.

The survey was also distributed to MD/DO students in the U.S., as has been done in prior studies, 5,12 which does limit the generalizability of our findings to this important and growing group of EM trainees.^36^ Additionally, the survey did not ask applicants who did not identify as non-LGBTQIA+ about whether or how LGBTQIA+ factors may have influenced their choice of residency rank, if at all. These applicants may have loved ones who do identify as LGBTQIA+ or may be allies. Future studies should distribute all survey questions to all applicants.

This study captured data from a single application cycle and was limited to EM, limiting its generalizability beyond the specialty. While we distributed our survey to approximately 75% of applicants, there exists the possibility of selection bias.^37^ Low response rates are a common challenge in surveys of medical trainees and raise concerns about nonresponse bias that may affect representativeness.^38^ To address this, best practices emphasize both transparent reporting of response rates and the use of standardized definitions such as those outlined by the American Association for Public Opinion Research, and explicit evaluation of nonresponse bias where feasible.^39^,^40^

While we based our study on earlier work and focused on factors that may influence LGBTQIA+ trainee residency preferences, we acknowledge that residency selection is an individual journey and that no survey in isolation could fully describe the factors that shape this journey. Also, program rank was limited to programs where applicants interviewed, which may have introduced selection bias since the interview invitations are influenced by factors including geographic preferences and application strategy.

Our use of state-level categorization may not have captured intrastate heterogeneity, since urban centers often differ from rural areas in their political climates and LGBTQIA+ community presence. The survey question assessing “political environment” may also be interpreted in many ways; respondents may have evaluated institutional, local, or state political contexts differently. Finally, we did not explicitly explore gender identity, which is distinct from sexual orientation. While LGBTQIA+ is often used as an umbrella term, it does not fully capture the nuanced experiences of trans and nonbinary individuals, who face unique social and policy challenges. Future initiatives should aim to better differentiate gender identity from sexual orientation to ensure more comprehensive representation and analysis.

## CONCLUSION

This study highlights the unique residency preferences and disclosure patterns observed in a convenience sample of LGBTQIA+ applicants in emergency medicine. Compared to non–LGBTQIA+ peers, they placed greater importance on program diversity and commitment to underserved communities, and less on proximity to partners or program length. LGBTQIA+ applicants tended to rank programs in states with more LGBTQIA+–friendly legislation more highly, although this pattern was nuanced and influenced by factors beyond legislation alone. While most supported mechanisms for disclosing LGBTQIA+ status, a significant minority did not, reflecting the nuanced considerations that influence disclosure decisions. Programs that cultivate inclusivity and align with these values are well-positioned to attract and support a diverse applicant pool.

## Supplementary Information











## Figures and Tables

**Table 1:** Demographic information of survey respondents in a study to determine how LGBTQIA+ identities influence residency selection.

	Number of respondents	Proportion
Race		
White	307	69.5%
Black	24	5.4%
American Indian/Alaskan Native	3	0.7%
Asian	61	13.8%
Multiracial	11	2.5%
Other	14	3.2%
Prefer not to answer	22	5.0%
Hispanic/Latino		
Yes	43	9.7%
No	391	88.3%
Prefer not to answer	9	2.0%
Gender identity		
Cisgender man	173	38.9%
Cisgender woman	254	57.1%
Transgender man	1	0.2%
Transgender woman	1	0.2%
Non-binary	4	1.0%
Genderqueer	1	0.2%
Unlisted Response* (written-in responses included 2 “male,” 1 “man,” and 1 “woman”)	4	1.0%
Prefer not to answer	7	1.6%
Sexual orientation		
Heterosexual/straight	365	82.2%
Gay/lesbian	14	3.2%
Bisexual	35	7.9%
Queer	12	2.7%
Pansexual	3	0.7%
Prefer not to answer	15	3.4%
Marital Status		
Committed partner/married	146	32.9%
Divorced	4	1.0%
Widowed	1	0.2%
Single/never married	284	64%
Prefer not to answer	9	2.0%
Non-traditional student		
Yes	194	43.7%
No	244	55%
Prefer not to answer	6	1.4%

**Table 2:** Comparison of the importance of specific residency factors between LGBTQIA+ and non-LGBTQIA+ respondents using chi-square test of proportions.

Factor	Proportion considered “important” among LGBTQIA+ trainees % (95% CI for proportion)	Proportion considered “important” among non-LGBTQIA+ trainees % (95% CI for proportion)	*P* value
Program length	66.1% (52.1–78.2)	80.3% (75.6–84.3)	.02
Geographical location	96.4% (87.7–99.6)	96.1% (93.5–97.8)	.90
Proximity to partner	64.3% (50.4–76.6)	80.8% (76.2–84.6)	< .01
Cost of living	57.1% (43.2–70.3)	69.4% (64.3–74.1)	.07
Program type (e.g., academic vs community)	92.9% (82.7–98.0)	90.0% (86.3–92.8)	.49
Program reputation	89.3% (78.1–96.0)	93.8% (90.8–96.1)	.21
Diversity within program	91.1% (80.4–97.0)	72.2% (67.2–76.8)	< .01
Commitment to underserved patient population	96.4% (87.7–99.6)	81.0% (76.5–84.9)	< .01
Interview day experience	92.9% (82.7–98.0)	95.2% (92.5–97.2)	.45
Experience with residents	92.9% (82.7–98.0)	96.1% (93.5–97.8)	.27
Experience with faculty	92.9% (82.7–98.0)	97.5% (95.2–98.8)	.07
