# Supplementary material for: Through the Prism: Shining Light on LGBTQIA+ Applicant Identities and Influences
Source: West J Emerg Med. 2026 May 18;27(3):698–708. doi: 10.5811/westjem.50598 (PMC13246176; doi:10.5811/westjem.50598)
Supplement: Supplementary file 2 [file wjem-27-698-s002.pdf]

# Factors Influencing Program Choice for Emergency Medicine-Bound Medical Students

How old are you?

[ ]

What is your gender identity?

- ○ Cis Man
- ○ Cis Woman
- ○ Transgender Man
- ○ Transgender Woman
- ○ Non-binary
- ○ Genderqueer
- ○ Two-spirit
- ○ [ ] If not listed or more than one, please specify:
- ○ Prefer not to answer

What is your sexual orientation?

- ○ Heterosexual/straight
- ○ Gay/Lesbian

- Bisexual
- Queer
- Pansexual
- Not Listed, please specify:
- Prefer not to answer

Are you a member of a population that is underrepresented in medicine?

(AAMC definition: African Americans and/or Black, Hispanic/Latino, Native American (American Indians, Alaska Natives, and Native Hawaiians), Pacific Islander, and mainland Puerto Rican OR from a disadvantaged background)

- Yes
- No
- Prefer not to answer

What is your race?

- White/Caucasian
- Black or African American
- American Indian or Alaskan Native
- Asian
- Native Hawaiian or other Pacific Islander

- ○ [ ] Multiracial (please specify)
- ○ [ ] Other (please specify)
- ○ Prefer not to answer

Are you of Hispanic/Latino origin?

- ○ Yes
- ○ No
- ○ Prefer not to answer

What is your marital status?

- ○ Committed partner/married
- ○ Divorced
- ○ Widowed
- ○ Separated
- ○ Single (never married)
- ○ Prefer not to answer

Are you a non-traditional applicant? (e.g. 2nd career, late entry, other degrees)

- ○ Yes
- ○ No

- ○ Prefer not to answer

Do you identify as LGBTQIA+?

- ○ Yes
- ○ No
- ○ Prefer not to answer

Did you disclose your LGBTQIA+ status in your application or interview?

- ○ Yes - Application
- ○ Yes - Interview
- ○ Yes - Both Interview and Applciation
- ○ No

Where in your application did you disclose your LGBTQIA+ status?
(Select all that apply)

- ☐ Designated pronouns
- ☐ Personal statement
- ☐ Experiences
- ☐ Hobbies
- ☐ ________ Other (please specify)

During what proportion of your interviews did you disclose your LGBTQIA+ status?

- None of them
- Some of them
- All of them

What factors led you to disclose your LGBTQIA+ status to some programs and not all programs?

Would you want the option of adding LGBTQIA+ status to the standard residency application?

- Yes
- No

If your medical school is located within the United States, in which state is it located?

What is the location of your first-ranked program?

What is the location of your second-ranked program?

What is the location of your third-ranked program?

How important were the following in creating your rank list?

| | Extremely important | Very important | Moderately important | Slightly important | Not at all important |
|---|---|---|---|---|---|
| Program length (3 vs 4 years) | ○ | ○ | ○ | ○ | ○ |
| Geographic location | ○ | ○ | ○ | ○ | ○ |
| Proximity to partner or family (including couples matching) | ○ | ○ | ○ | ○ | ○ |
| Cost of living | ○ | ○ | ○ | ○ | ○ |

| | Extremely important | Very important | Moderately important | Slightly important | Not at all important |
|---|---|---|---|---|---|
| Program type (academic vs community) | ○ | ○ | ○ | ○ | ○ |
| Program reputation | ○ | ○ | ○ | ○ | ○ |
| Diversity within the program (residents & faculty) | ○ | ○ | ○ | ○ | ○ |
| Program commitment to the underserved community | ○ | ○ | ○ | ○ | ○ |
| Interview day experience | ○ | ○ | ○ | ○ | ○ |
| Experience with residents | ○ | ○ | ○ | ○ | ○ |
| Experience with faculty | ○ | ○ | ○ | ○ | ○ |

Which choice describes the relative importance of location versus program characteristics in your decision of where to rank programs?

- ○ Completely based on individual program characteristics
- ○ Primarily based on program characteristics, but location played a role
- ○ Equal balance between program characteristics and location
- ○ Primarily based on location, but program characteristics played a role

○ Completely based on location

How important were the following in creating your rank list?

| | Extremely important | Very important | Moderately important | Slightly important | Not at all important |
|---|---|---|---|---|---|
| Friendliness of location to LGBTQIA+ | ○ | ○ | ○ | ○ | ○ |
| Friendliness of health system/hospital to LGBTQIA+ | ○ | ○ | ○ | ○ | ○ |
| Friendliness of residency to LGBTQIA+ | ○ | ○ | ○ | ○ | ○ |
| LGBTQIA+ residents in the program | ○ | ○ | ○ | ○ | ○ |
| LGBTQIA+ faculty in the faculty | ○ | ○ | ○ | ○ | ○ |
| LGBTQIA+ educational curriculum | ○ | ○ | ○ | ○ | ○ |
| Political environment | ○ | ○ | ○ | ○ | ○ |
| Presence/absence of anti-LGBTQIA+ laws | ○ | ○ | ○ | ○ | ○ |

Powered by Qualtrics
