# Supplementary material for: Through the Prism: Shining Light on LGBTQIA+ Applicant Identities and Influences
Source: West J Emerg Med. 2026 May 18;27(3):698–708. doi: 10.5811/westjem.50598 (PMC13246176; doi:10.5811/westjem.50598)

Supplement 3. The original response distributions of the importance of LGBTQIA+-related factors for rank list creation prior to dichotomization.


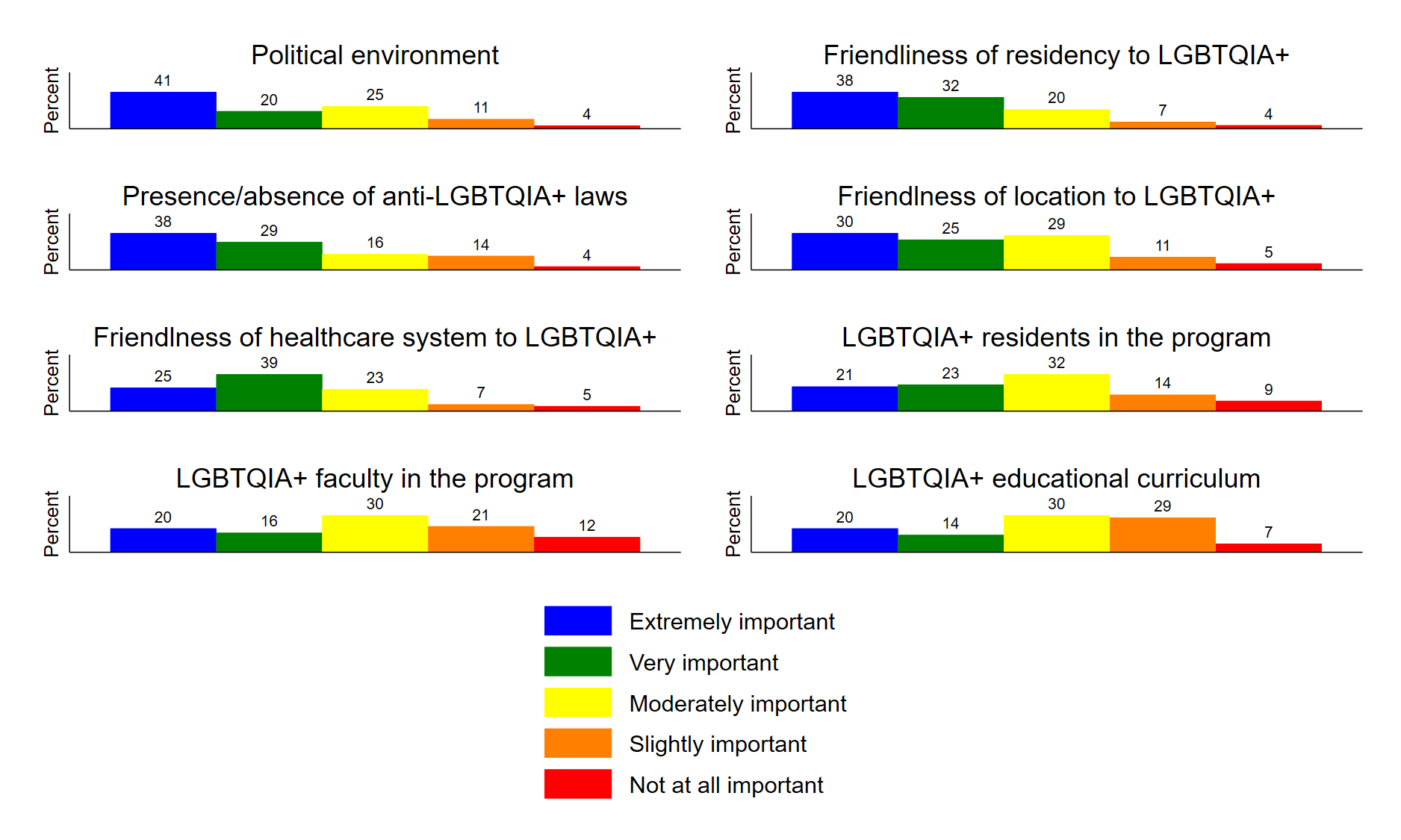

Supplement: Supplementary file 3 [file wjem-27-698-s003.docx]
