# Supplementary material for: Through the Prism: Shining Light on LGBTQIA+ Applicant Identities and Influences
Source: West J Emerg Med. 2026 May 18;27(3):698–708. doi: 10.5811/westjem.50598 (PMC13246176; doi:10.5811/westjem.50598)

Supplement 4. The original response distributions for comparison of residency factors between LGBTQIA+ and non-LGBTQIA+ respondents prior to dichotomization.
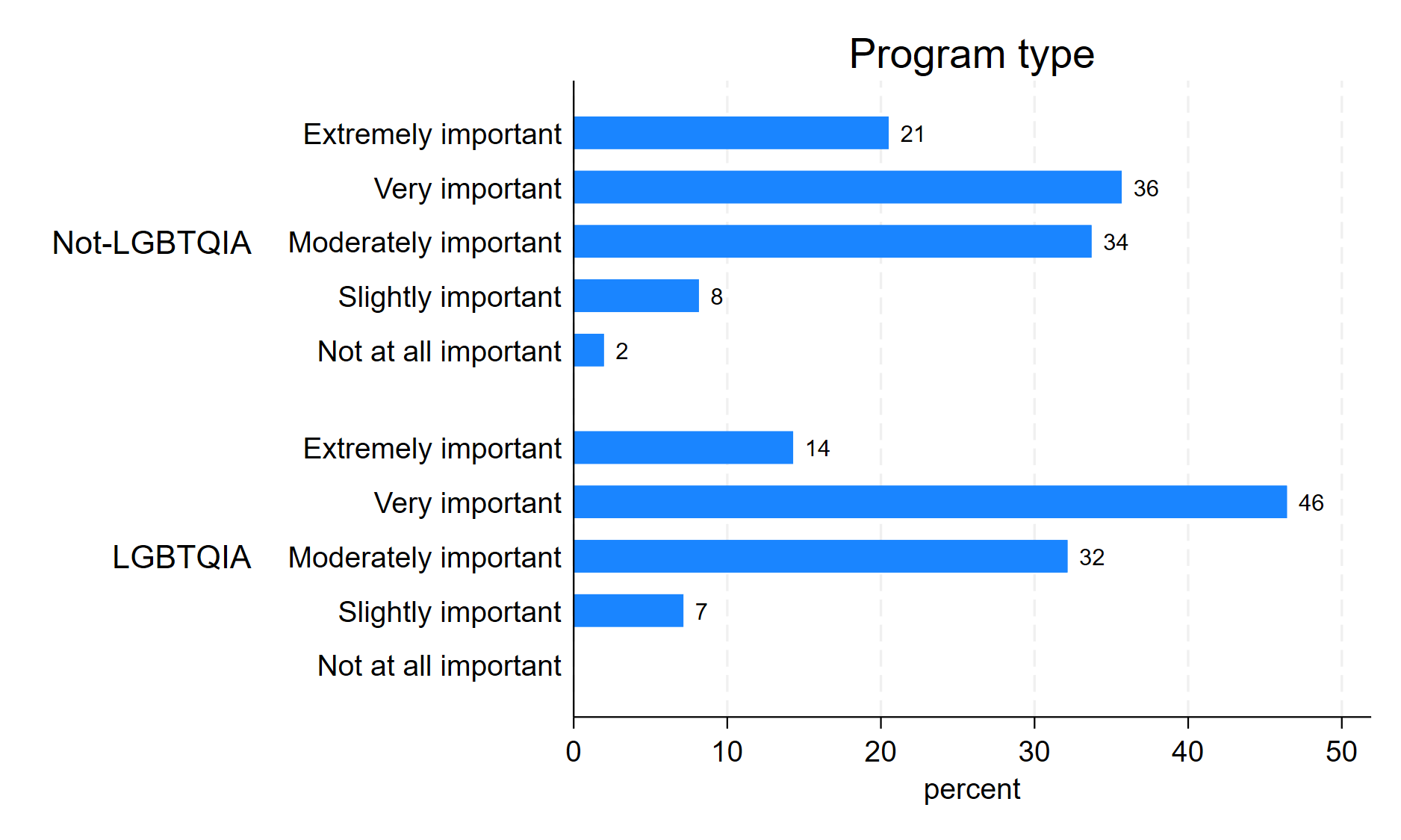

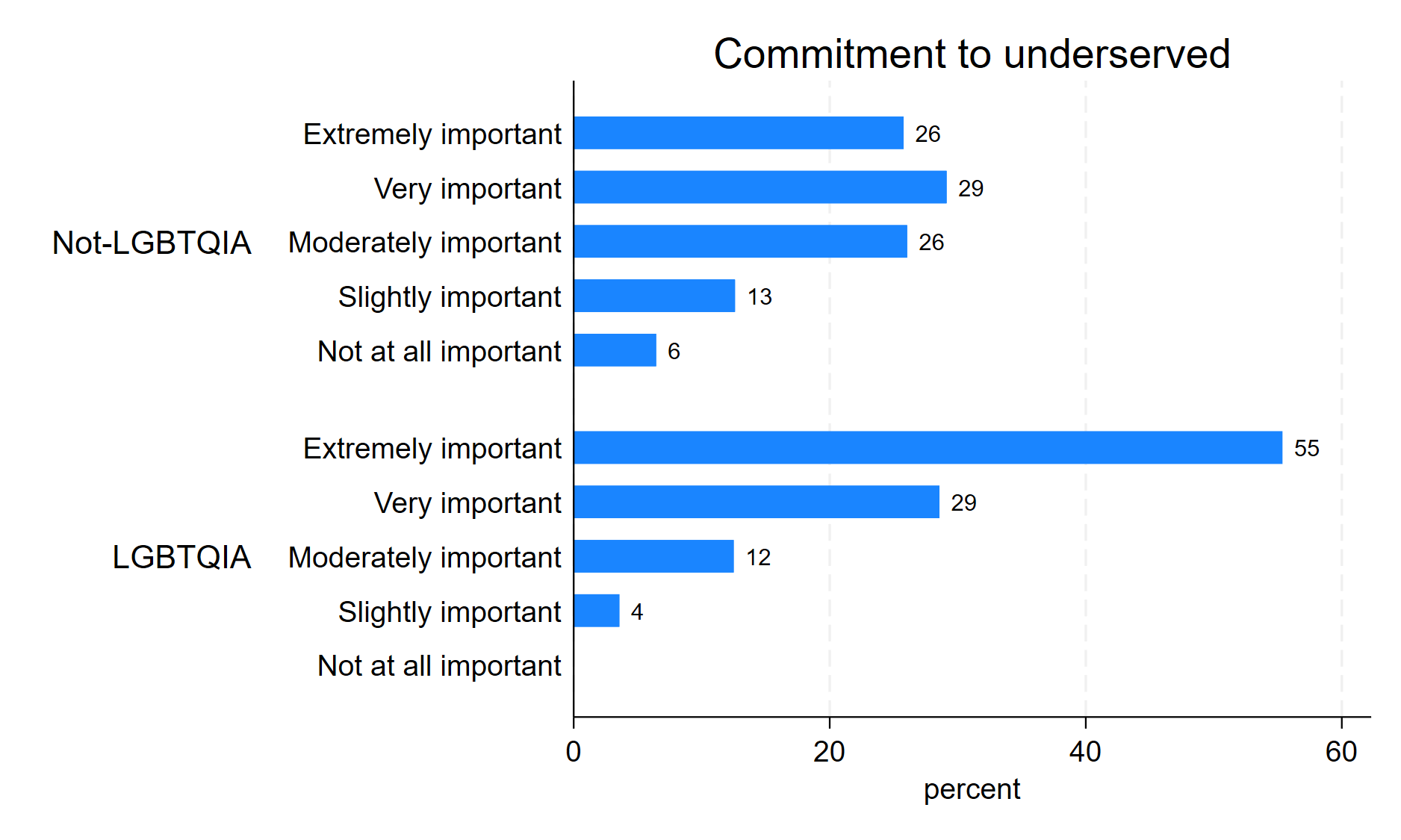

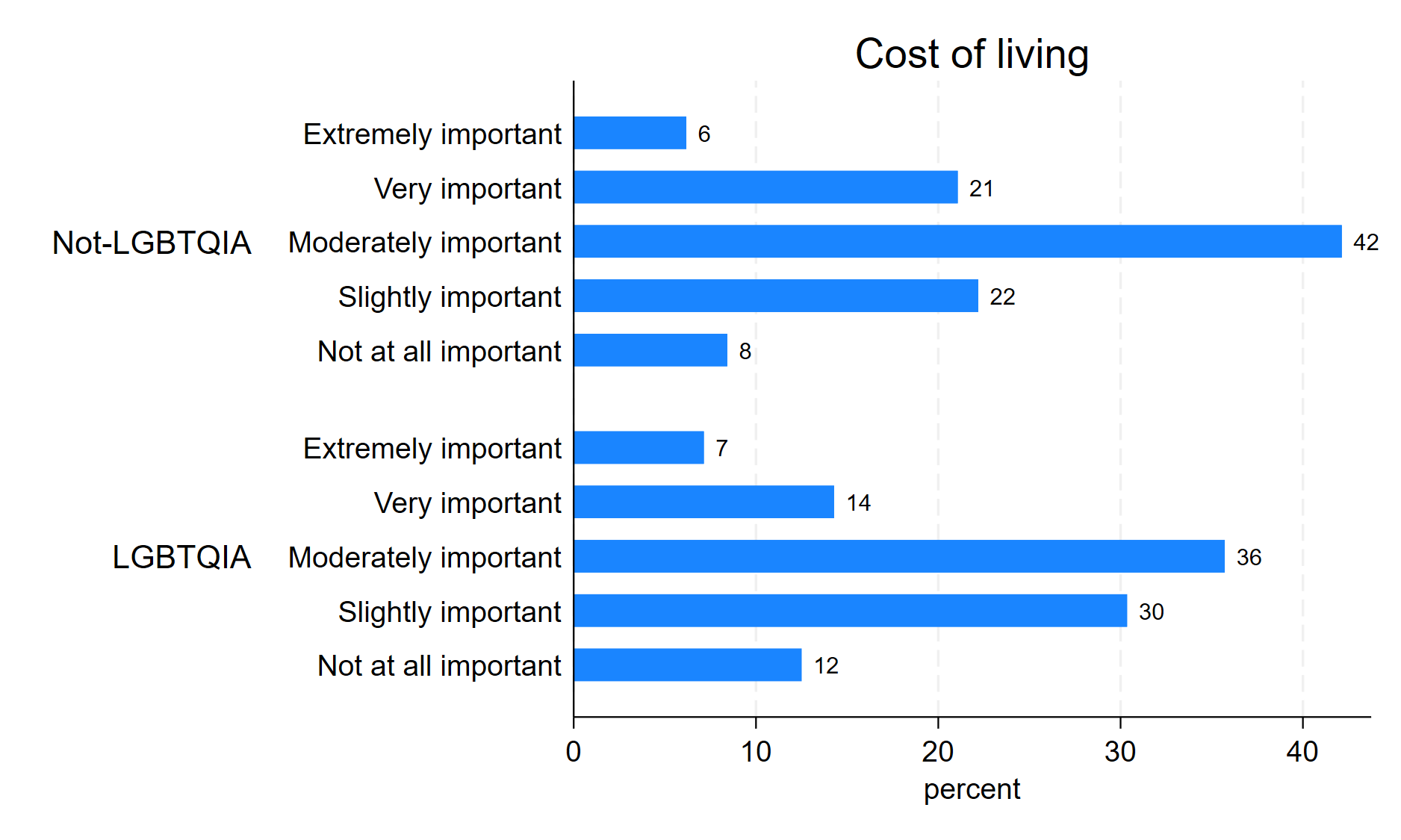

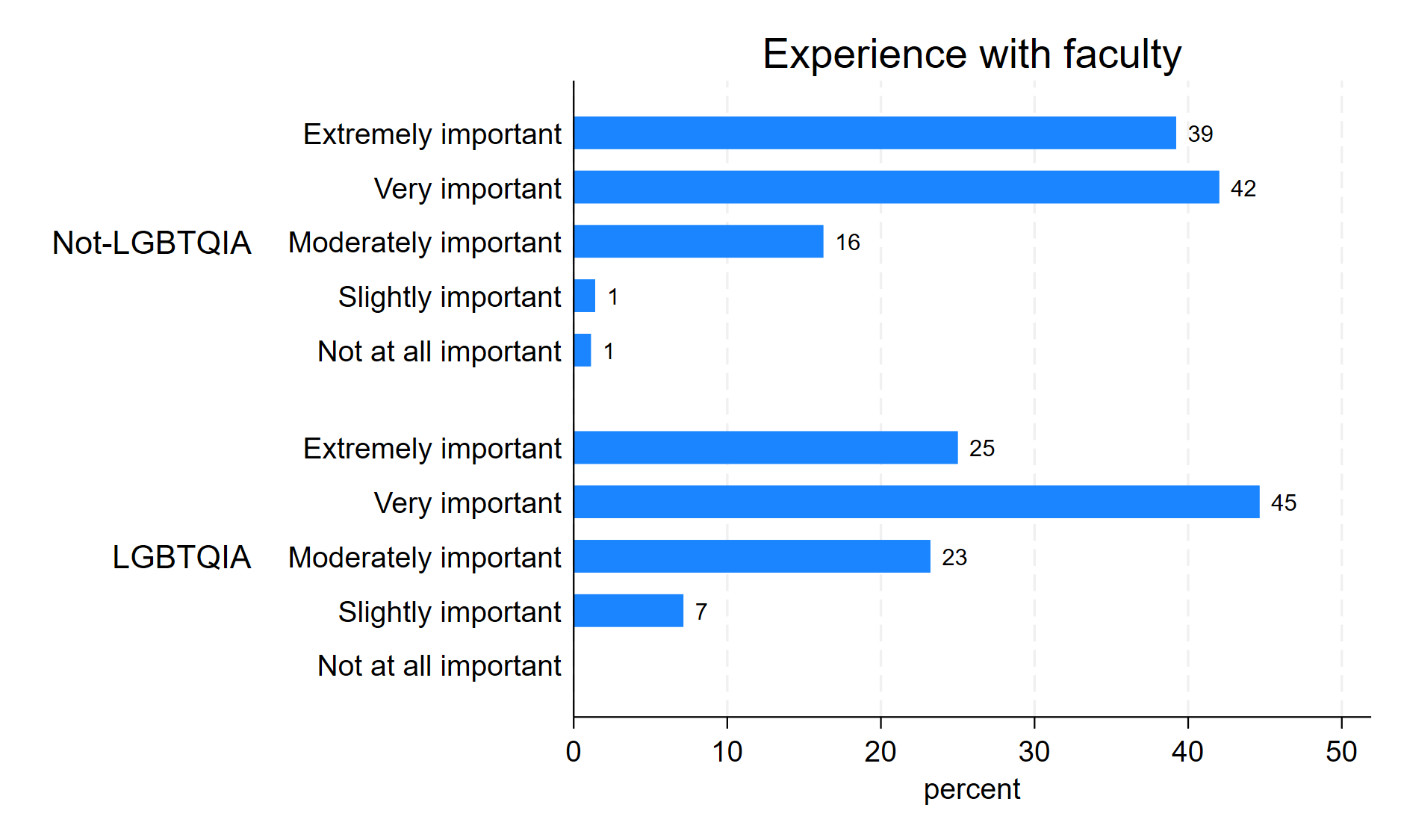

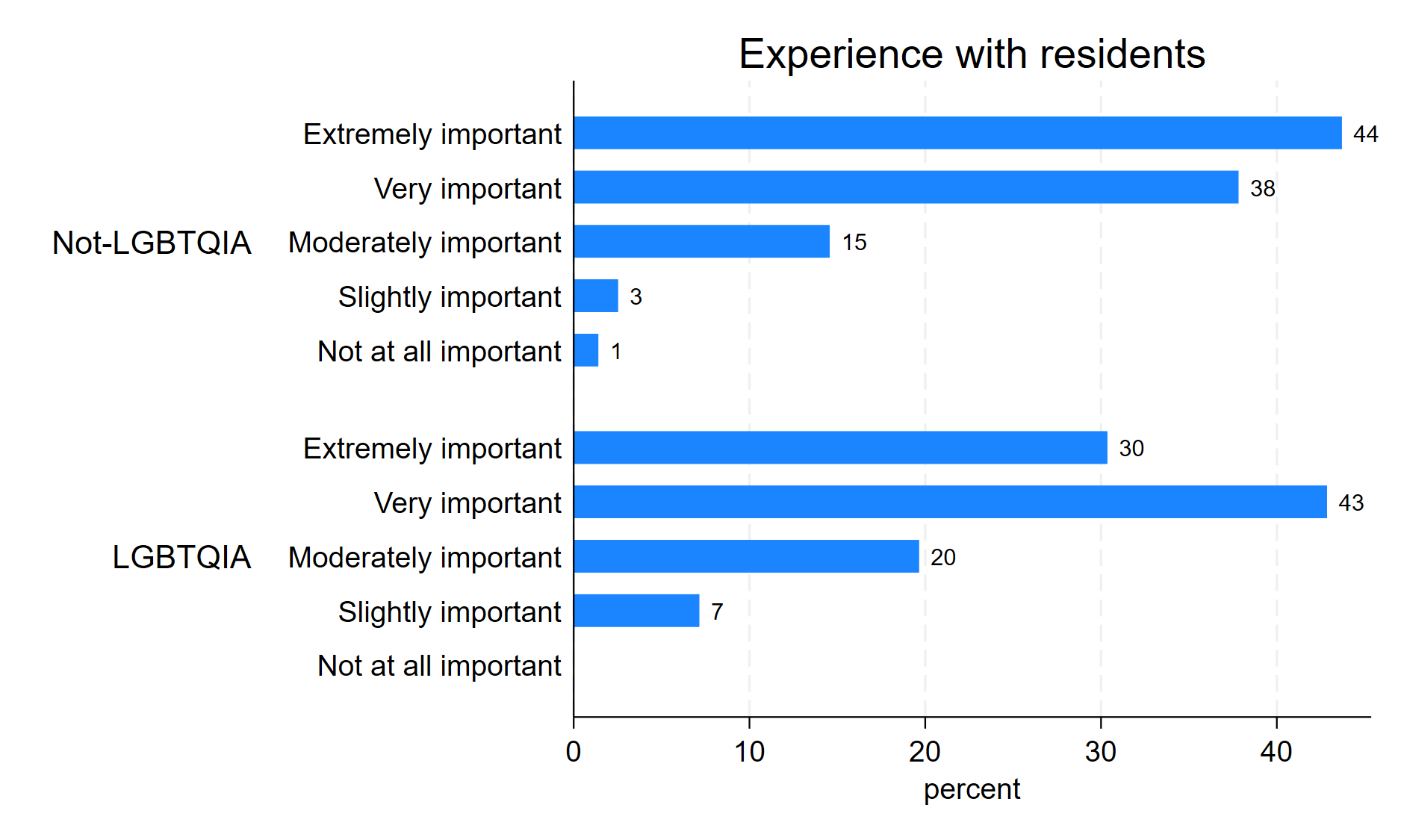

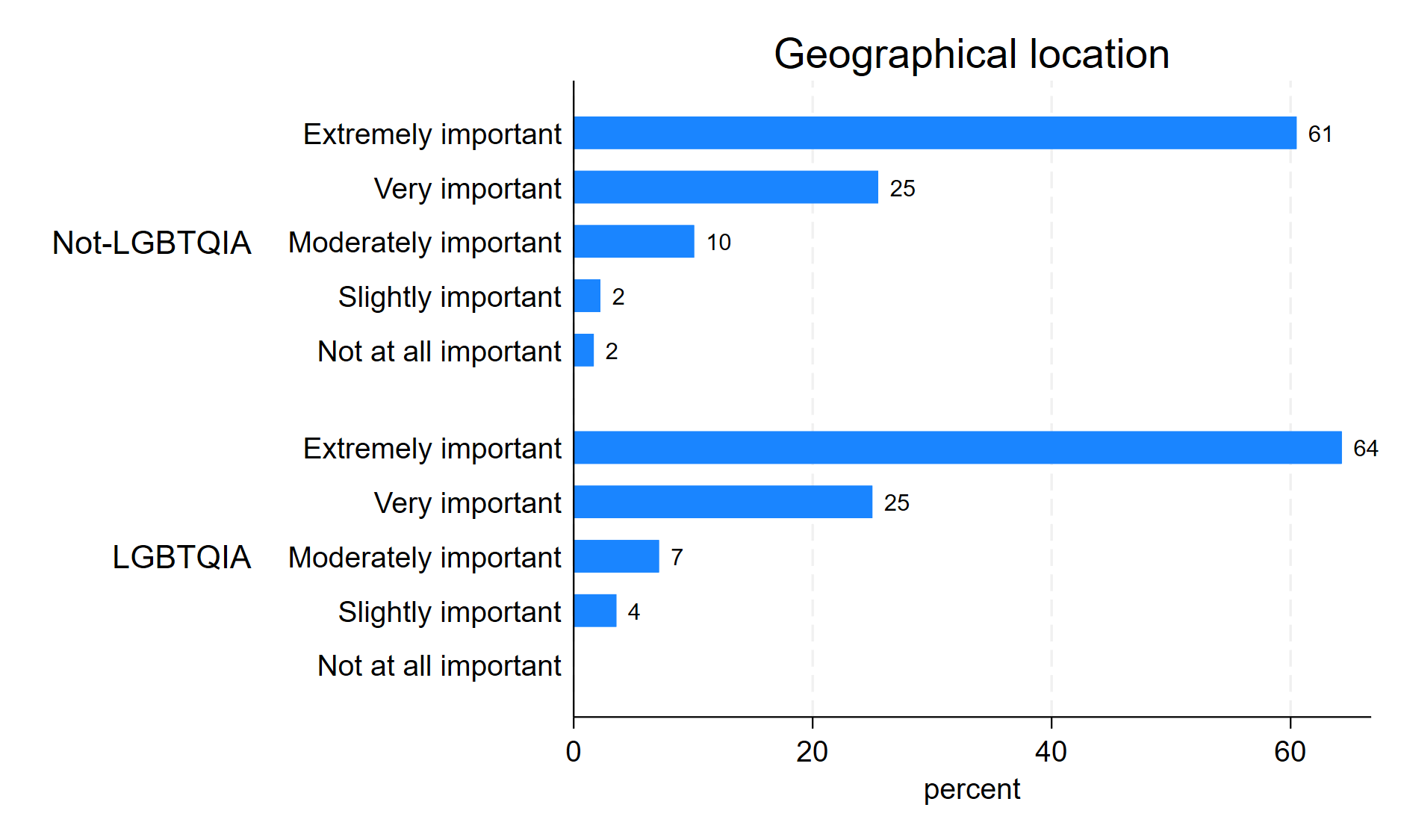

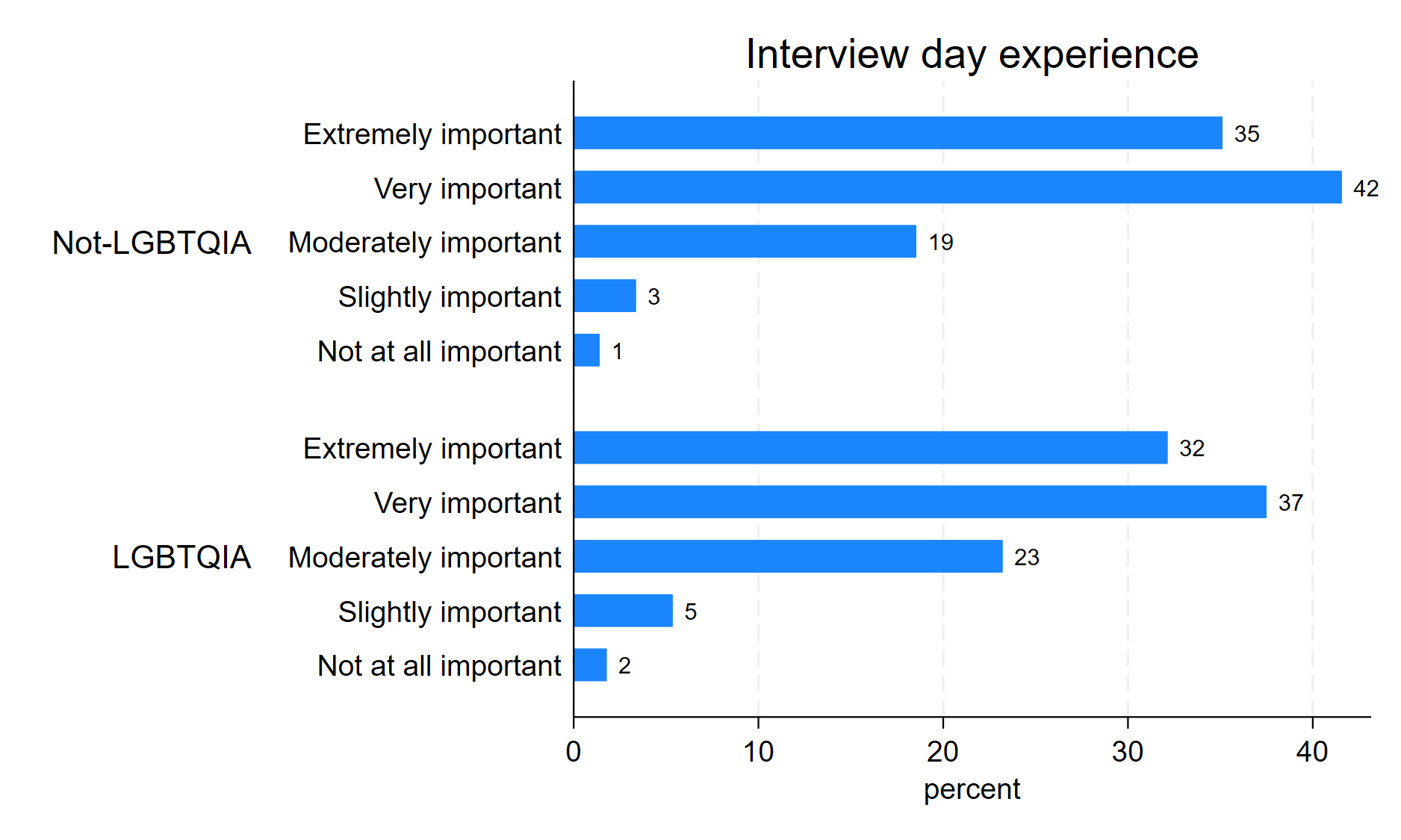

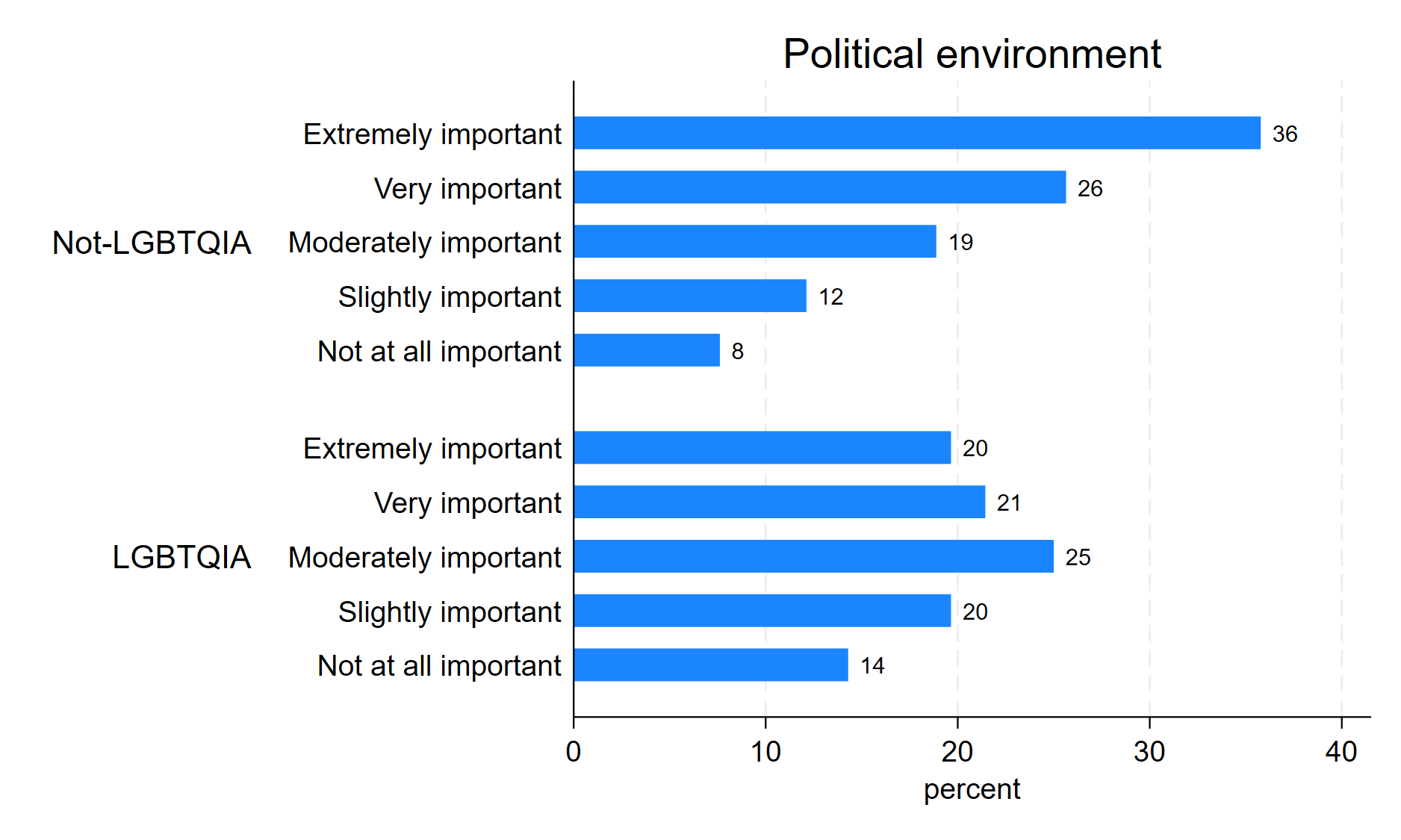

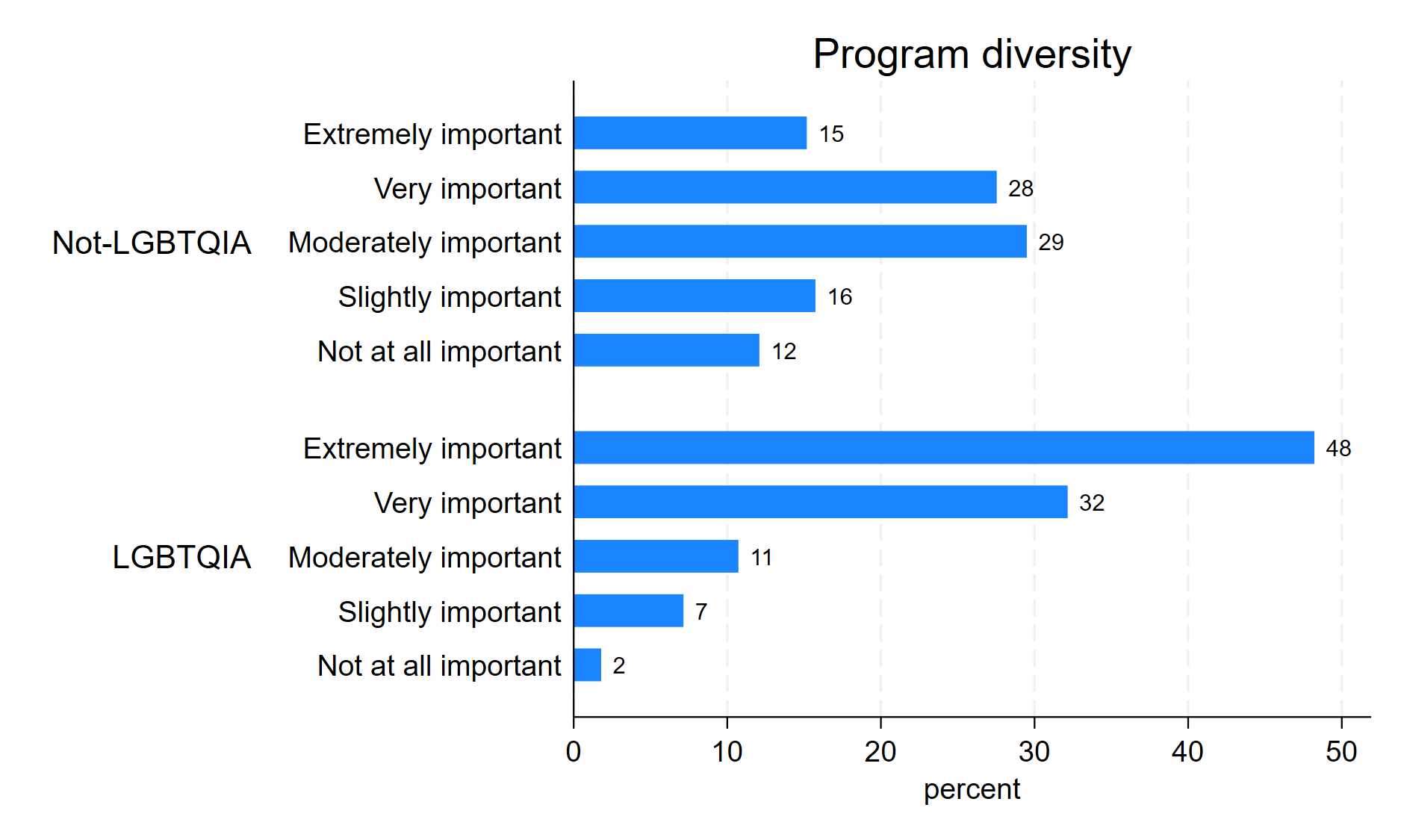

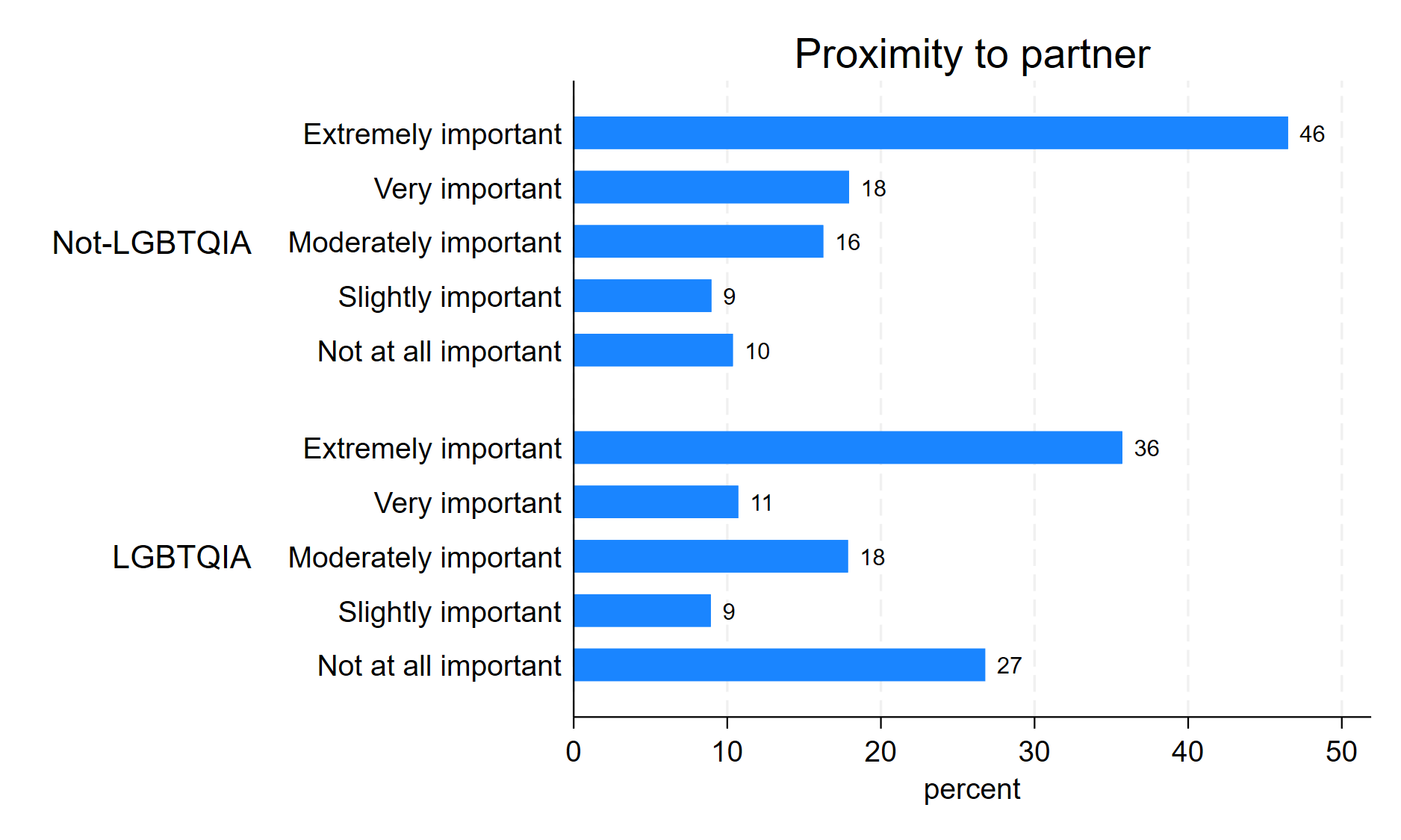

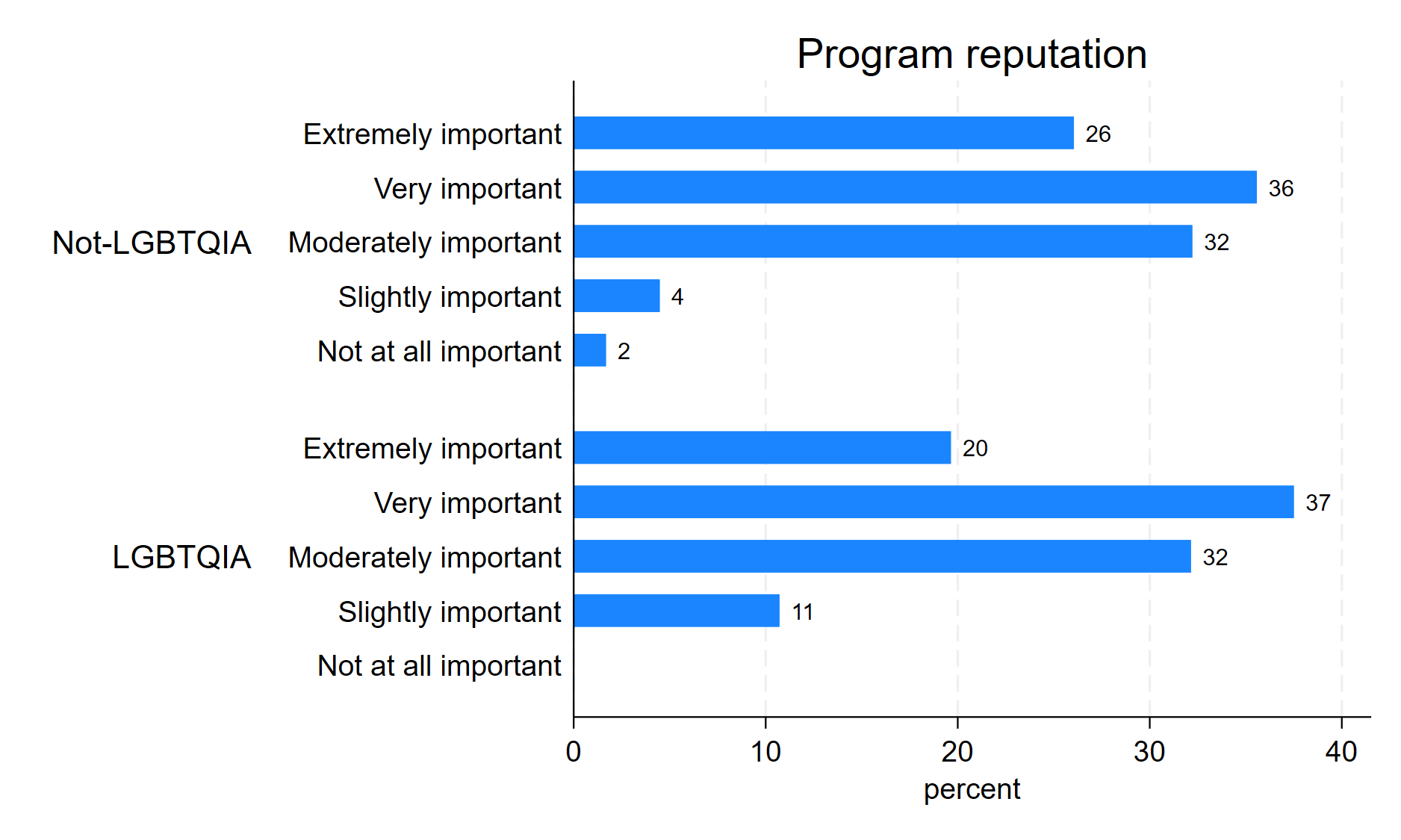

Supplement: Supplementary file 4 [file wjem-27-698-s004.docx]
