# Supplementary material for: Through the Prism: Shining Light on LGBTQIA+ Applicant Identities and Influences
Source: West J Emerg Med. 2026 May 18;27(3):698–708. doi: 10.5811/westjem.50598 (PMC13246176; doi:10.5811/westjem.50598)
Supplement: Supplementary file 5 [file wjem-27-698-s005.docx]

Supplement 5, Table 1. Nonresponse bias wave analysis of demographic characteristics.

| Demographic Characteristic | Subgroup | Early wave | Late waves | p-value |
| --- | --- | --- | --- | --- |
| Age (IQR) | - | 28 (27, 30) | 28 (26, 29) | 0.021 |
| Gender |  |  |  | 0.49 |
|  | Cis Man | 77 (42.8%) | 99 (37.4%) |  |
|  | Cis Woman | 96 (53.3%) | 159 (60.0%) |  |
|  | Transgender Man | 1 (0.6%) | 0 (0.0%) |  |
|  | Transgender Woman | 1 (0.6%) | 0 (0.0%) |  |
|  | Non-binary | 2 (1.1%) | 2 (0.8%) |  |
|  | Genderqueer | 0 (0.0%) | 1 (0.4%) |  |
|  | Prefer not to answer | 3 (1.7%) | 4 (1.5%) |  |
| Marital status |  |  |  | 0.48 |
|  | Committed partner/married | 67 (37.2%) | 79 (29.9%) |  |
|  | Divorced | 2 (1.1%) | 2 (0.8%) |  |
|  | Widowed | 0 (0.0%) | 1 (0.4%) |  |
|  | Single (never married) | 108 (60.0%) | 176 (66.7%) |  |
|  | Prefer not to answer | 3 (1.7%) | 6 (2.3%) |  |
| Race |  |  |  | 0.57 |
|  | White | 129 (72.1%) | 178 (67.7%) |  |
|  | Black or African American | 6 (3.4%) | 18 (6.8%) |  |
|  | American Indian or Alaskan Native | 1 (0.6%) | 2 (0.8%) |  |
|  | Asian | 23 (12.8%) | 38 (14.4%) |  |
|  | Multiracial | 4 (2.2%) | 7 (2.7%) |  |
|  | Other | 8 (4.5%) | 6 (2.3%) |  |
|  | Prefer not to answer | 8 (4.5%) | 14 (5.3%) |  |
| Sexual Orientation |  |  |  | 0.98 |
|  | LGBTQIA+ | 24 (13.8%) | 35 (13.7%) |  |
|  | Non-LGBTQIA+ | 150 (86.2%) | 220 (86.3%) |  |
| Ethnicity |  |  |  | 0.85 |
|  | Hispanic/Latine | 19 (10.6%) | 24 (9.1%) |  |
|  | Not Hispanic/Latine | 157 (87.2%) | 234 (89.0%) |  |
|  | Prefer not to answer | 4 (2.2%) | 5 (1.9%) |  |

Early wave is defined as the first release of the survey on May 16, 2024. Late waves are defined by response after a reminder email prompt to complete the survey (between May 28, 2024 and June 24, 2024). P-value was done with null hypothesis that there is no difference in proportions of categorical variables or using the Wilcoxon rank-sum test for continuous variables (age)

Supplement 5, Table 2. Nonresponse bias wave analysis of importance of specific residency factors.

|  | Early wave | | Late waves | | Interaction p-value |
| --- | --- | --- | --- | --- | --- |
|  | Not LGBTQIA+ | LGBTQIA+ | Not LGBTQIA+ | LGBTQIA+ |  |
| Program length | 78.4% | 60.9% | 81.6% | 69.7% | 0.77 |
| Geographical location | 93.9% | 95.7% | 97.6% | 96.7% | 0.70 |
| Proximity to partner | 77.7% | 69.6% | 82.8% | 60.6% | 0.26 |
| Cost of living | 68.0% | 56.5% | 70.3% | 57.6% | 0.91 |
| Program type | 90.5% | 100.0% | 89.5% | 87.9% | 0.99 |
| Program reputation | 91.2% | 82.6% | 95.7% | 93.9% | 0.68 |
| Diversity within program | 66.7% | 87.0% | 76.1% | 93.9% | 0.70 |
| Commitment to underserved patient population | 75.7% | 100.0% | 84.7% | 93.9% | 0.99 |
| Interview day experience | 95.2% | 87.0% | 95.2% | 97.0% | 0.22 |
| Experience with residents | 93.9% | 91.3% | 97.6% | 93.9% | 0.62 |

Early wave is defined as the first release of the survey on May 16, 2024. Late waves are defined by response after a reminder email prompt to complete the survey (between May 28, 2024 and June 24, 2024). Interaction terms between LGBTQIA+ status and the importance of residency factors were analyzed with the p-value shown for the interaction term between early vs late waves of respondents * LGBTQIA+ status.
